# Point-of-care testing reduces antibiotic prescribing for adults with community-acquired pneumonia: a systematic review and meta-analysis

**DOI:** 10.3389/fphar.2026.1728667

**Published:** 2026-04-29

**Authors:** Wenshuai Ji, Kai Xie, Kang Zhang, Yingjin Liang, Yikun Wang, Hao Yang, Yifan Han, Wenjing Yin, Haifeng Wang

**Affiliations:** 1 Department of Respiratory Medicine, The First Affiliated Hospital of Henan University of Chinese Medicine, Zhengzhou, China; 2 Academy of Chinese Medical Sciences, The First Clinical Medical College of Henan University of Chinese Medicine, Zhengzhou, China; 3 Co-Construction Collaborative Innovation Center for Chinese Medicine and Respiratory Diseases by Henan & Education Ministry of P. R. China, Henan University of Chinese Medicine, Zhengzhou, China

**Keywords:** antibiotics, community-acquired pneumonia, meta-analysis, point-of-care testing, procalcitonin

## Abstract

**Objectives:**

To evaluate the impact of point-of-care testing (POCT) on antibiotic prescribing and clinical outcomes in adult patients with community-acquired pneumonia (CAP) through a systematic review and meta-analysis.

**Methods:**

Web of Science, Scopus, Embase, PubMed, and Cochrane Library were searched for randomized controlled trials (RCTs). The final search was conducted on June 2025. Outcomes included both antibiotic-related and clinical outcomes. The meta-analysis was performed using Review Manager. The study was registered with PROSPERO (CRD420251035572).

**Results:**

Nine RCTs involving 2,899 participants were included. POCT significantly reduced the proportion of adult CAP patients receiving antibiotic prescriptions by 10% (RD = −0.10; 95% CI: −0.15 to −0.05, *P* < 0.0001) and shortened the duration of antibiotic treatment by 2.68 days (MD = −2.68; 95% CI: −4.41 to −0.95, *P* = 0.002). Available evidence suggests no signal of harm, although safety outcomes were reported in relatively few trials, such as the length of hospital stay (MD = −0.43; 95% CI: −1.41 to 0.54, *P* = 0.38). Sensitivity analyses confirmed the robustness of these findings.

**Conclusion:**

POCT may reduce antibiotic exposure in patients with CAP without compromising clinical outcomes. As diagnostic innovation plays an increasingly important role in combating antimicrobial resistance, POCT should be used selectively as an adjunct to clinical judgment.

## Introduction

Community-acquired pneumonia (CAP) refers to an infectious disease of the lung parenchyma acquired outside hospital settings and may lead to severe complications such as acute lung injury, acute respiratory distress syndrome, and septic shock (Capelastegui et al., 2014; Serrano et al., 2022). Globally, CAP remains one of the leading causes of mortality, accounting for approximately 3 million deaths worldwide annually (Organization WH, n.d.). It poses a serious threat to public health and imposes a substantial burden on healthcare systems and societal resources (Jain et al., 2015; Aliberti et al., 2016; Ramirez et al., 2017). In the United States alone, CAP results in approximately 1.4 million emergency department visits, 740,000 hospitalizations, and 41,000 deaths each year, with annual hospitalization costs reaching $7.7 billion (Vaughn et al., 2024). Among critically ill patients requiring intensive care, the 30-day mortality is 27%, increasing to 47% at 1 year (Liapikou, 2020).

Antibiotic therapy constitutes the cornerstone of CAP treatment. While a substantial proportion of CAP cases are viral in etiology, empirical broad-spectrum antibiotic therapy is frequently initiated due to concerns about bacterial coinfection or superinfection, compounded by the low yield of conventional microbiological cultures and resulting diagnostic uncertainty (Braykov et al., 2014; Kang et al., 2021). Although clinical guidelines recommend tailoring antibiotic regimens based on pathogen type, disease severity, and specific agents, with treatment durations generally ranging from 5 to 21 days (Braykov et al., 2014; Wiersinga et al., 2018), real-world practice frequently diverges. Driven by concerns over treatment failure or recurrence—especially in patients with severe comorbidities, critical illness, or poor therapeutic response—clinicians often extend therapy durations or engage in antibiotic overuse (Aliberti et al., 2015; Uranga et al., 2016; Schweitzer et al., 2022). Such inappropriate and excessive antibiotic prescribing not only fails to improve patient outcomes in many cases but also heightens the risks of adverse drug reactions and antimicrobial resistance. Concurrently, shifts in pathogen distribution, fueled by population aging and the rise of immunocompromised populations, have contributed to an increasing prevalence of resistant organisms (Torres et al., 2015).

Point-of-care testing (POCT), a diagnostic approach that delivers rapid results at the site of patient care, holds promise in guiding initial treatment decisions and antibiotic use in CAP (Price, 2001; Palomeque et al., 2024). Biomarkers such as procalcitonin (PCT) and C-reactive protein (CRP), which increase significantly during bacterial infections, can aid in distinguishing bacterial from non-bacterial etiologies and inform initiation or discontinuation of antibiotics (Van Duffel et al., 2022). Other POCT platforms, including molecular diagnostics, enable more precise pathogen identification, facilitating personalized antimicrobial therapy and reducing unnecessary use of broad-spectrum antibiotics (Poole et al., 2022). The integration of POCT into CAP management may enhance diagnostic accuracy, refine antibiotic prescribing strategies, and play a critical role in curbing the escalating threat of antimicrobial resistance.

The relentless rise of antimicrobial resistance underscores the urgent global need for more judicious antibiotic stewardship (GBD, 2021 Antimicrobial Resistance Collaborators, 2024). In the context of CAP, striking a balance between effective treatment and prudent antibiotic use remains a pressing clinical challenge. POCT, with its speed and convenience, has shown promise as a decision-support tool in this regard (Palomeque et al., 2024). Previous studies have shown that POCT, such as CRP and PCT, can reduce antibiotic use in patients with acute respiratory tract infections (ARTIs) (Schuetz et al., 2018; Zhang et al., 2022). However, these studies have primarily focused on specific biomarkers and have not specifically targeted CAP. Therefore, we conducted a systematic review and meta-analysis that specifically evaluates the effectiveness and safety of POCT in guiding antibiotic management for adult patients with CAP, integrating multiple POCT modalities and assessing both antibiotic-related and clinical outcomes.

## Methods

Our systematic review and meta-analysis was conducted in accordance with the Preferred Reporting Items for Systematic Reviews and Meta-Analyses (PRISMA) 2020 guidelines and the Cochrane Handbook (Version 6.5) (Higgins et al., 2019; Page et al., 2021). The study protocol was prospectively registered on the International Prospective Register of Systematic Reviews (PROSPERO: CRD420251035572).

### Search strategy

Two reviewers independently performed a comprehensive literature search across five major databases: Web of Science, Scopus, Embase, PubMed, and the Cochrane Library. The search encompassed all records from database inception to June 2025. Only English-language studies involving adult participants were included. A combination of Medical Subject Headings (MeSH) and relevant free-text terms was employed, structured around three core concepts: CAP, POCT, and antibiotics. The full search strategy is detailed in Supplementary Material 1. We included randomized controlled trials enrolling adults with CAP or mixed lower respiratory tract infection populations, provided that CAP patients constituted the full study population or a clearly extractable and clinically relevant subgroup.

### Selection criteria

We included RCTs published in English that evaluated the effectiveness of POCT in guiding antibiotic prescribing for adult patients (aged ≥18 years) with a confirmed diagnosis of CAP, compared with usual care, defined as standard clinical management without POCT, typically guided by evidence-based guidelines or the treating physician’s judgment, with routine laboratory tests performed as needed. POCT was defined as any diagnostic test performed at or near the site of patient care, providing rapid results that could inform clinical decision-making and potentially improve patient outcomes (Drain et al., 2014), regardless of the specific technology or method used.

Studies were excluded if they were preprints, review articles (systematic or narrative), letters, editorials, study protocols, conference abstracts, or trial registry records. We also excluded studies without full-text availability, duplicate publications, cluster-randomized trials, and those involving pediatric populations.

Two reviewers (WS Ji and K Xie) independently screened titles, abstracts, and full texts using Rayyan (https://www.rayyan.ai/) based on the eligibility criteria. Discrepancies were resolved through discussion or consultation with a third reviewer (K Zhang).

### Outcome measures

Antibiotic-related outcomes included: (1) antibiotic prescription rate, defined as the proportion of patients who received antibiotics; (2) duration of antibiotic treatment; and (3) cost of antibiotics per patient. Clinical outcomes included: (1) length of hospital stay; (2) intensive care unit (ICU) transfer rate; (3) 30-day all-cause mortality; (4) 30-day all-cause readmission; and (5) composite adverse events rate, defined as the occurrence of one or more negative outcomes such as antibiotic-related side effects, secondary bacterial infections, worsening of clinical symptoms, or disease-specific complications.

### Data extraction

Two reviewers (WS Ji and H Yang) independently extracted data and evaluated the methodological and clinical content of each included study. Discrepancies were resolved through discussion, with arbitration by a third reviewer (WJ Yin) when necessary. Extracted data included: first author, year of publication, country, study period, patient demographics (age and sex), sample size, intervention characteristics (type of POCT, timing of testing, adherence to POCT-guided recommendations, and duration of observation), follow-up duration, and reported outcomes.

### Quality assessment

Two reviewers (YJ Liang and YF Han) independently assessed the risk of bias using the Risk of Bias Tool 2 (RoB 2) (Sterne et al., 2019), covering five domains: randomization, deviations from interventions, missing data, outcome measurement, and selective reporting. Each domain was rated as low risk, some concerns, or high risk. Disagreements were resolved through discussion with a third reviewer (K Zhang).

### Data analysis

Meta-analyses were performed using Review Manager (RevMan, version 5.4; Cochrane Collaboration). For continuous outcomes, results were expressed as mean differences (MD) or standardized mean differences (SMD) with corresponding 95% confidence intervals (CI). Dichotomous outcomes were summarized using risk differences (RD) with 95% CI. Here, RD represents the absolute difference in event rates between groups, CI indicates the confidence interval around the effect estimate, and *I* ^2^ quantifies the proportion of variability due to between-study heterogeneity rather than chance. Heterogeneity was assessed using the chi-squared (χ^2^) test and quantified by the *I* ^*2*^ statistic. A fixed-effects model was applied when *I* ^*2*^ was ≤50%, while a random-effects model was used when *I* ^*2*^ exceeded 50%. For studies reporting medians with interquartile ranges or ranges, means and standard deviations were estimated using the method proposed by McGrath et al. (2020). Sensitivity and subgroup analyses were conducted to explore potential sources of heterogeneity.

### Role of the funding source

The funder had no role in the design or conduct of the study; collection, management, analysis, or interpretation of the data; or preparation, review, or approval of the manuscript.

## Results

### Study selection

**FIGURE 1:**
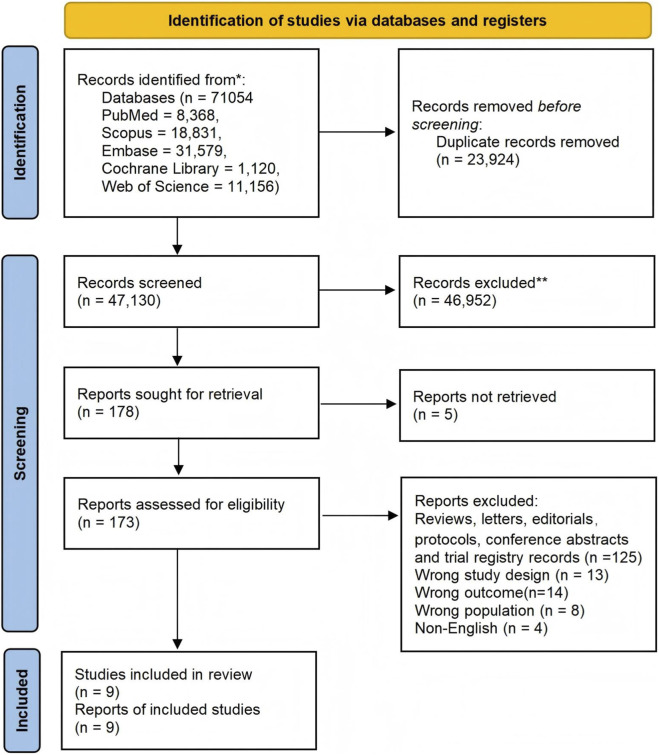
PRISMA flow diagram of study selection.

The initial literature search yielded 71,054 records. After removing duplicates, 47,130 unique records remained. Title and abstract screening led to the exclusion of 46,952 records, and 178 full-text articles were sought for retrieval. Of these, five were not retrieved, and 164 were excluded for reasons including study design and language. Ultimately, nine RCTs involving a total of 2,899 participants met the inclusion criteria and were included in the final meta-analysis. The complete study selection process is summarized in the PRISMA flow diagram (Figure 1).

### Characteristics of the included studies

Among the nine included randomized controlled trials (see reference list in Supplementary Material 2), four studies exclusively enrolled patients with CAP, while another four studies included patients with lower respiratory tract infections. The remaining study recruited patients with ARTIs but provided a clearly defined subset of CAP cases suitable for analysis.

The types of POCT intervention were distributed as follows: 7 studies used PCT, and 2 studies used molecular POCT. The testing timing of POCT in 8 studies was between 5 min and 2 h, and 1 study more than 2 h (Supplementary Material 3).

Among the nine included studies, POCT was employed solely to guide antibiotic initiation in one study, antibiotic discontinuation in another, and both initiation and discontinuation in seven studies. All seven studies utilizing PCT-based protocols adopted broadly similar algorithms: antibiotic therapy was discouraged when PCT concentrations were <0.25 μg/L and encouraged when levels exceeded this threshold. No explicit antibiotic algorithm was reported in the two studies using molecular POCT platforms.

### Risk of bias assessment

All included RCTs were appraised with the Cochrane RoB 2.0 tool (Supplementary Material 4). All trials reported appropriate methods of random sequence generation, indicating a low risk of selection bias. Due to the nature of POCT, blinding of participants and personnel was generally not feasible, which may have introduced performance bias. However, seven studies implemented blinded outcome assessment and were therefore judged to be at low risk of detection bias. In addition, all trials were assessed as having a low risk of selective reporting, and most studies maintained attrition and loss to follow-up rates within acceptable limits.

### Antibiotic prescription rate

**FIGURE 2:**
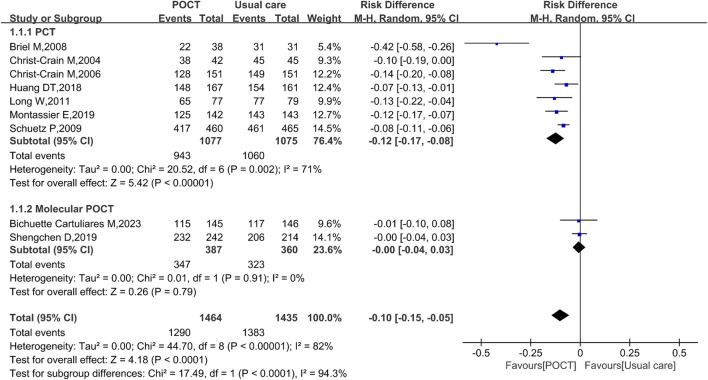
Random-effects forest plot of the effect of POCT intervention on antibiotic prescribing rate by POCT type. M–H: Mantel–Haenszel method; df: degrees of freedom; RD: risk difference; CI: confidence intervals.

Nine studies, enrolling a total of 2,899 participants, reported data on the antibiotic prescription rate. Pooled analysis showed that POCT was associated with an approximately 10% reduction in the antibiotic prescription rate among patients with CAP (RD = −0.10, 95% CI: −0.15 to −0.05, *I* ^*2*^ = 82%, *P* < 0.0001). Specifically, PCT - guided strategies resulted in a 12% decrease in antibiotic prescriptions (RD = −0.12, 95% CI: −0.17 to −0.08, *I* ^*2*^ = 71%, *P* < 0.00001). In contrast, no significant difference was observed between molecular POCT and usual care (RD = −0.00, 95% CI: −0.04 to 0.03, *I* ^*2*^ = 0%, *P* = 0.79) (Figure 2).

### Duration of antibiotic treatment

**FIGURE 3:**
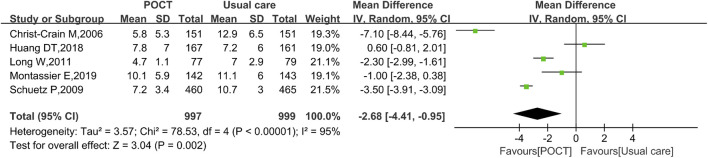
Random-effects forest plot of the effect of POCT intervention on duration of antibiotic treatment. M–H: Mantel–Haenszel method; df: degrees of freedom; MD: mean differences; CI: confidence intervals.

Five studies evaluating the duration of antibiotic treatment, comprising 1,996 participants, demonstrated that the POCT group exhibited a statistically significant reduction in antibiotic treatment duration by 2.68 days compared to usual care (MD = −2.68, 95% CI: −4.41 to −0.95, *I* ^*2*^ = 95%, *P* = 0.002) (Figure 3).

### Cost of antibiotics per patient

**FIGURE 4:**
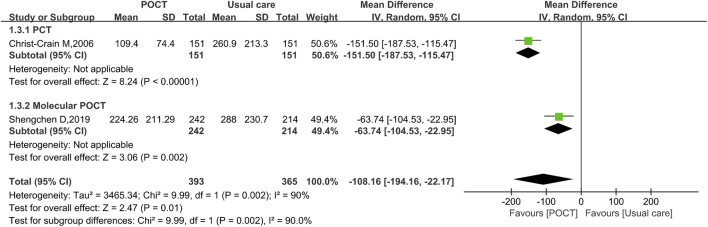
Random-effects forest plot of the effect of POCT intervention on cost of antibiotics per patient by POCT type. M–H: Mantel–Haenszel method; df: degrees of freedom; MD: mean differences; CI: confidence intervals.

Two studies assessing the cost of antibiotics per patient, involving 758 participants, revealed that the POCT group demonstrated a statistically significant reduction in antibiotic treatment costs compared to usual care (MD = −108.16, 95% CI: −194.16 to −22.17, *I* ^*2*^ = 90%, *P* = 0.01). In subgroup analyses, both the PCT subgroup (MD = −151.50, 95% CI = −187.53 to −115.47, *P* < 0.00001) and molecular POCT subgroup (MD = −63.74, 95% CI = −104.53 to −22.95, *P* = 0.002) showed significant reductions in cost of antibiotics per patient, aligning with the overall pooled effect (Figure 4).

### Length of hospital stay

**FIGURE 5:**
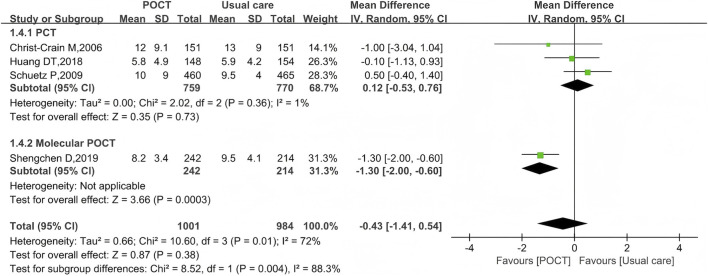
Random-effects forest plot of the effect of POCT intervention on length of hospital stay by POCT type. M–H: Mantel–Haenszel method; df: degrees of freedom MD: mean differences; CI: confidence intervals.

Length of hospital stay was reported in four trials including a total of 1,985 participants. The mean duration ranged from 5.8 to 12 days in the POCT groups and from 5.9 to 13 days in the usual care groups. Pooled analysis showed no statistically significant difference between the two groups (MD = −0.43, 95% CI: −1.41 to 0.54, *I* ^*2*^ = 72%, *P* = 0.38). A single study involving molecular POCT demonstrated a statistically significant reduction in hospital stay (MD = −1.30, 95% CI: −2.00 to −0.60, *P* = 0.0003), while the pooled analysis of studies using PCT-based POCT showed no significant difference compared to usual care (MD = 0.12; 95% CI: −0.53 to 0.76, *I* ^*2*^ = 1%, *P* = 0.73) (Figure 5).

### ICU transfer rate

**FIGURE 6:**
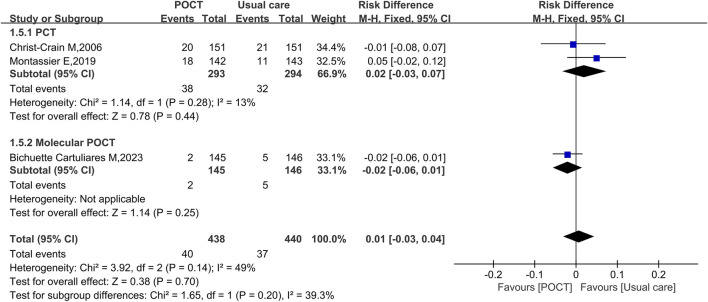
Fixed-effects forest plot of the effect of POCT intervention on ICU transfer rate by POCT type. M–H: Mantel–Haenszel method; df: degrees of freedom; RD: risk difference; CI: confidence intervals.

Three studies evaluating the ICU transfer rate, including 878 participants, indicated no significant difference in ICU transfer rates between the POCT group and usual care (RD = 0.01, 95% CI: −0.03 to 0.04, *I* ^*2*^ = 49%, *P* = 0.70). In the PCT-based POCT subgroup, the difference remained non-significant (RD = 0.02; 95% CI: −0.03 to 0.07, *I* ^*2*^ = 13%, *P* = 0.44). Similarly, the molecular POCT subgroup did not show a significant effect (RD = −0.02, 95% CI: −0.06 to 0.01, *P* = 0.25) (Figure 6).

### 30-day all-cause mortality

**FIGURE 7:**
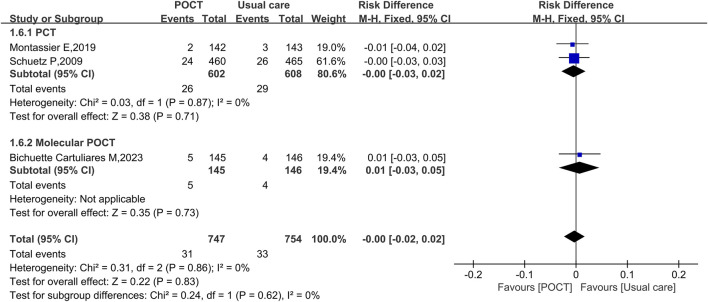
Fixed-effects forest plot of the effect of POCT intervention on 30-day all-cause mortality by POCT type. M–H: Mantel–Haenszel method; df: degrees of freedom; RD: risk difference; CI: confidence intervals.

Three studies assessing 30-day all-cause mortality, involving a total of 1,501 participants, reported no statistically significant difference between the POCT group and the usual care group (RD = −0.00, 95% CI: −0.02 to 0.02, *I* ^*2*^ = 0%, *P* = 0.83). Subgroup analyses confirmed consistent results across POCT types: the PCT subgroup (RD = −0.00, 95% CI: −0.03 to 0.02, *I* ^*2*^ = 0%, *P* = 0.71) and molecular POCT subgroup (RD = 0.01, 95% CI: −0.03 to 0.05, *P* = 0.73) both demonstrated no significant mortality difference, suggesting that POCT modality did not affect the outcome (Figure 7).

### 30-day all-cause readmission

**FIGURE 8:**
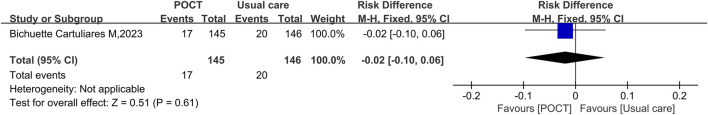
Fixed-effects forest plot of the effect of POCT intervention on 30-day all-cause readmission by POCT type. M–H: Mantel–Haenszel method; df: degrees of freedom; RD: risk difference; CI: confidence intervals.

One study involving 291 participants evaluated the all-cause readmission rate within 30 days of discharge. The findings showed no statistically significant difference between the POCT group and the usual care group (RD = −0.02, 95% CI: −0.10 to 0.06, *P* = 0.61) (Figure 8).

### Composite adverse events rate

**FIGURE 9:**
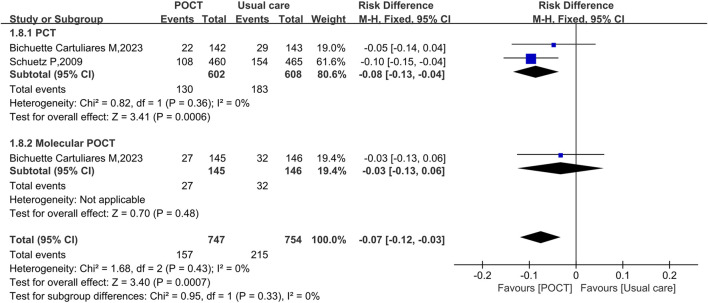
Fixed-effects forest plot of the effect of POCT intervention on composite adverse events rate by POCT type. M–H: Mantel–Haenszel method; df: degrees of freedom; RD: risk difference; CI: confidence intervals.

Three studies, enrolling a total of 1,501 participants, reported data on the composite adverse events rate. Through pooled analysis, it was revealed that POCT was associated with an approximately 7% reduction in the composite adverse events rate among patients with CAP (RD = −0.07, 95% CI: −0.12 to −0.03, *I* ^*2*^ = 0%, *P* = 0.0007). Specifically, PCT - guided strategies resulted in 8% decrease in composite adverse events (RD = −0.08, 95% CI: −0.13 to −0.04, *I* ^*2*^ = 0%, *P* = 0.0006). In contrast, no significant difference was observed between the molecular POCT group and the usual care group (RD = −0.03, 95% CI: −0.13 to 0.06, *P* = 0.48) (Figure 9).

### Sensitivity analysis

To evaluate the robustness of the meta-analysis findings, sensitivity analyses were performed by sequentially removing individual studies for outcomes with high heterogeneity. The results indicated that exclusion of any single study did not materially alter the direction or statistical significance of the pooled effect estimates, suggesting good overall stability. Removal of certain studies led to slight reductions in the *I^2^* statistic, but the impact on heterogeneity was limited, indicating that no individual study had a dominant influence on the overall results. In addition, reanalysis of dichotomous outcomes using risk ratios instead of risk differences did not change the direction of the findings (Supplementary Material 5).

## Discussion

Our study provides up-to-date evidence on the impact of POCT on antibiotic use and related clinical outcomes in adult patients with CAP. The findings suggest that POCT may play a pivotal role in promoting rational antibiotic prescribing, shortening treatment duration, and reducing associated costs. Notably, even when antimicrobial therapy had already been initiated before POCT results were available, early discontinuation was still feasible, leading to shorter overall treatment courses. Importantly, this reduction in antibiotic exposure was not associated with worse clinical outcomes, including length of stay, ICU admission, mortality, or all-cause readmission.

CAP is a heterogeneous disease caused by a variety of pathogens, including bacteria and viruses. Respiratory viruses are detectable in approximately 10∼30% of hospitalized immunocompetent adults with CAP (Cillóniz et al., 2011). While antibiotics remain essential for treating bacterial infections, their unnecessary use contributes significantly to antimicrobial resistance (AMR) (Abbas et al., 2024). Previous studies have reported that up to 37.4% of patients with CAP receive at least one inappropriate antibiotic prescription (Ryu et al., 2024). Therefore, reducing non-essential antibiotic use without compromising clinical efficacy has become a central priority in contemporary medical practice. POCT facilitates this goal by enabling more accurate and timely decision-making, reducing diagnostic uncertainty, and curbing antibiotic overuse—an imperative in global efforts to combat AMR.

Our meta-analysis revealed that antibiotic prescription rates were significantly reduced by 10% in patients managed with POCT, with PCT-guided strategies accounting for a 12% reduction. In contrast, molecular POCT did not significantly reduce prescribing rates, likely reflecting its primary utility in pathogen identification rather than guiding treatment duration or necessity. POCT was also associated with shorter antibiotic courses, indicating improved appropriateness of initiation and more timely discontinuation.

Although data were limited, we observed a reduction in per-patient antibiotic-related costs in the POCT group. Despite upfront implementation expenses, POCT may offer potential long-term savings through more targeted and abbreviated antibiotic use (Boere et al., 2022). However, POCT did not significantly reduce hospital length of stay—a key driver of total healthcare costs—leaving the overall cost-effectiveness uncertain. Further studies are needed to comprehensively evaluate its economic impact.

Unlike its clear effect on antibiotic use, POCT did not demonstrate significant improvements in broader clinical outcomes such as length of stay, ICU transfers, 30-day mortality, or readmissions. Although one molecular POCT study reported a modest reduction in hospital stay, pooled estimates—particularly those based on PCT—showed no significant differences. These findings suggest that POCT may enhance antimicrobial stewardship without compromising the quality or safety of care. Notably, POCT was associated with a significant reduction in composite adverse events, further supporting the safety of de-escalation strategies guided by POCT. These results align with existing literature indicating that reduced antibiotic use under POCT guidance is safe (Kyriazopoulou et al., 2021).

Subgroup analyses indicated heterogeneity in the effects of different POCT modalities. PCT-guided strategies showed consistent benefits across most outcomes, whereas molecular POCT did not reduce antibiotic prescribing rates but was associated with shorter hospital stays, reduced readmissions, and fewer adverse events. These differences likely reflect their distinct mechanisms: PCT offers real-time insights into the host response, while molecular diagnostics focus on pathogen identification and typically do not provide actionable guidance on when to discontinue antibiotics.

Our findings are consistent with prior research. For instance, Schuetz et al. reported that PCT-based protocols reduced antibiotic exposure in patients with CAP without affecting mortality or treatment failure. Similarly, Berg and Lindhardt (2012) demonstrated reduced antibiotic use without increased risk using PCT algorithms. However, evidence on molecular POCT for guiding antibiotic therapy in CAP remains limited. A randomized controlled trial by Poole et al. (2022) showed that molecular testing for lower respiratory pathogens improved antibiotic de-escalation in ICU patients with pneumonia but had no significant effect on total antibiotic duration or clinical safety endpoints—possibly due to the high prevalence of viral–bacterial co-infections, which limits the utility of virus identification alone in guiding antibiotic cessation.

Clinicians generally hold favorable views toward POCT for managing lower respiratory tract infections, citing its role in enhancing diagnostic certainty—particularly through CRP/PCT thresholds or molecular detection—and in reducing empiric misprescribing. Most patients also accept POCT as part of routine care, which may help manage expectations regarding antibiotics (Wood et al., 2011). As clinicians continue to face pressure to balance timely treatment with responsible antimicrobial stewardship, POCT offers an evidence-based compromise.

However, the clinical impact of POCT depends not only on diagnostic accuracy but also on adherence to associated decision algorithms (Orwig et al., 2024). Health policymakers and institutional leaders play a key role in promoting and facilitating POCT implementation (Damschroder et al., 2009; Teebagy et al., 2022). Previous studies have reported wide variability in compliance, with clinicians occasionally overriding POCT results due to concerns over patient expectations, comorbidities, or potential clinical deterioration (Schols et al., 2018). To maximize its utility, POCT implementation should be supported by clear guidelines, clinician education, and integrated decision-support tools (Little et al., 2019).

Currently, evidence remains limited regarding the use of molecular or CRP-based POCT in CAP. The independent impact of viral detection on antibiotic use was not analyzed, as outcomes were not stratified by pathogen type, and molecular POCT results were interpreted as part of integrated diagnostic panels, particularly given the potential for viral–bacterial co-infections. Future head-to-head trials comparing POCT modalities across various care settings—such as emergency departments, primary care clinics, and rural hospitals—are needed. Additionally, the long-term impact of POCT on antimicrobial resistance trends and healthcare economics warrants further investigation.

This review has several limitations. First, certain outcomes, such as cost of antibiotics per patient and 30-day all-cause readmission, were derived from a small number of studies, which may have limited the precision of the findings. Second, significant heterogeneity was observed for some outcomes, such as treatment duration and length of stay, possibly reflecting differences in patient populations and healthcare settings, which may limit generalizability. Nevertheless, sensitivity analyses using both fixed- and random-effects models yielded consistent conclusions, enhancing the robustness of our findings. Third, most included studies were conducted in high-resource settings, limiting the applicability of findings to low- and middle-income countries, where POCT might offer even greater benefits. Finally, only English-language articles were included. Although we identified some non-English studies, they were unlikely to meet the inclusion criteria or alter the overall conclusions.

Despite these limitations, our findings suggest that POCT may be useful in guiding antibiotic therapy for CAP without evidence of worse clinical outcomes. These results indicate that POCT has the potential to support antimicrobial stewardship. To better understand its role, future studies should focus on improving adherence, evaluating its use across diverse clinical settings, and generating further evidence on its clinical and economic effectiveness.
