# Supplementary material for: Point-of-care testing reduces antibiotic prescribing for adults with community-acquired pneumonia: a systematic review and meta-analysis
Source: Front Pharmacol. 2026 Apr 29;17:1728667. doi: 10.3389/fphar.2026.1728667 (PMC13167562; doi:10.3389/fphar.2026.1728667)
Supplement: Supplementary file 1 [file Supplementaryfile1.docx]

**Supplementary materials**

**TABLE OF CONTENTS**

| Appendix (1): Complete search strategies. | Page: 1-10 |
| --- | --- |
| Appendix (2): the studies included in the meta-analysis | Page: 11 |
| Appendix (3): Characteristics of each study. | Page: 12-14 |
| Appendix (4): Risk of bias graph | Page: 15 |
| Appendix (5): Sensitivity analysis using RR for all outcomes. | Page: 15 |

## Appendix (1): Complete search strategies.

PubMed search strategy:

| Search Number | Search Details | Results |
| --- | --- | --- |
| #1 | pneumonia[MeSH Terms] | 402,512 |
| #2 | pneumonia[Title/Abstract] | 171,848 |
| #3 | CAP[Title/Abstract] | 58,639 |
| #4 | ((((lung*[Title/Abstract]) OR (pulmonary[Title/Abstract])) OR (pleur*[Title/Abstract])) OR (respiratory[Title/Abstract])) AND ((infect*[Title/Abstract]) OR (inflam*[Title/Abstract])) | 431.265 |
| #5 | #1 OR #2 OR #3 OR #4 | 884,832 |
| #6 | Point-of-Care Testing[MeSH Terms] | 5,422 |
| #7 | Point-of-Care Systems[MeSH Terms] | 22,713 |
| #8 | (("point-of-care"[Title/Abstract] OR "near patient"[Title/Abstract] OR poc[Title/Abstract] OR rapid[Title/Abstract] OR bedside[Title/Abstract]) AND (test*[Title/Abstract] OR diagnostic*[Title/Abstract] OR analys*[Title/Abstract] OR system*[Title/Abstract] OR comput*[Title/Abstract] OR immunoassay*[Title/Abstract] OR techn*[Title/Abstract] OR immunofluorescence[Title/Abstract] OR "fluorescent antibody"[Title/Abstract] OR classif*[Title/Abstract] OR detect*[Title/Abstract] OR differenti*[Title/Abstract] OR "polymerase chain reaction"[Title/Abstract] OR pcr[Title/Abstract] OR naat[Title/Abstract] OR "molecular diagnostic*"[Title/Abstract] OR "nucleic acid amplification"[Title/Abstract] OR microarray[Title/Abstract] OR antigen*[Title/Abstract] OR radiography[Title/Abstract] OR ultraso*[Title/Abstract] OR x-ray[Title/Abstract] OR tomography[Title/Abstract])) | 670,070 |
| #9 | Biomarkers[MeSH Terms] | 956,810 |
| #10 | ((biomarker*[Title/Abstract]) AND (biological[Title/Abstract])) AND ((marker*[Title/Abstract]) OR (indicator*[Title/Abstract])) | 13,004 |
| #11 | Procalcitonin[MeSH Terms] | 2,420 |
| #12 | ((calcitonin*[Title/Abstract]) OR (procalcitonin*[Title/Abstract])) OR (pct[Title/Abstract]) | 46,340 |
| #13 | C-Reactive Protein[MeSH Terms] | 58,903 |
| #14 | (c reactive protein[Title/Abstract]) OR (crp[Title/Abstract]) | 127,207 |
| #15 | Molecular Diagnostic Techniques[MeSH Terms] | 22,723 |
| #16 | #6 OR #7 OR #8 OR #9 OR #10 OR #11 OR #12 OR #13 OR #14 | 1,764,678 |
| #17 | Anti-Bacterial Agents[MeSH Terms] | 493,782 |
| #18 | Anti-Infective Agents[MeSH Terms] | 902,510 |
| #19 | Antimicrobial Stewardship[MeSH Terms] | 4,247 |
| #20 | antibiotic*[Title/Abstract] OR amoxicillin*[Title/Abstract] OR penicillin*[Title/Abstract] OR macrolide*[Title/Abstract] OR beta-lactam*[Title/Abstract] OR tetracyclin*[Title/Abstract] OR quinolone*[Title/Abstract] OR ciprofloxacin*[Title/Abstract] | 610,915 |
| #21 | ("anti-bacterial"[Title/Abstract] OR antibacterial[Title/Abstract] OR bacteriocid*[Title/Abstract] OR "anti-infective"[Title/Abstract] OR antiinfective[Title/Abstract] OR "anti-mycobacterial"[Title/Abstract] OR antimycobacterial[Title/Abstract] OR antimicrobial[Title/Abstract]) AND (agent*[Title/Abstract] OR compound*[Title/Abstract] OR stewardship[Title/Abstract] OR drug*[Title/Abstract] OR combin*[Title/Abstract] OR prescri*[Title/Abstract] OR therapy[Title/Abstract] OR treat*[Title/Abstract]) | 265,338 |
| #22 | #17 OR #18 OR #19 OR #20 OR #21 | 1,302,629 |
| #23 | #5 OR #16 OR #22 | 8,368 |

Scopus search strategy:

| Search Number | Search Details | Results |
| --- | --- | --- |
| #1 | TITLE-ABS-KEY ( pneumonia ) | 560,910 |
| #2 | TITLE-ABS-KEY ( cap ) | 161,636 |
| #3 | TITLE-ABS-KEY (( lung* OR pulmonary OR pleur* OR respiratory ) W/4 ( infect* OR inflam* )) | 325,236 |
| #4 | #1 OR #2 OR #3 | 955,096 |
| #5 | TITLE-ABS-KEY ( ( "point-of-care" OR "near patient" OR poc OR rapid OR bedside ) W/5 ( test* OR diagnostic* OR analys* OR system* OR comput* OR immunoassay* OR techn* OR immunofluorescence OR "fluorescent antibody" OR classif* OR detect* OR differenti* OR "polymerase chain reaction" OR pcr OR naat OR "molecular diagnostic*" OR "nucleic acid amplification" OR microarray OR antigen* OR radiography OR ultraso* OR x-ray OR tomography ) ) | 451,226 |
| #6 | TITLE-ABS-KEY ( biomarker* ) | 784,884 |
| #7 | TITLE-ABS-KEY ( biological W/3 ( marker* OR indicator* ) ) | 628,925 |
| #8 | TITLE-ABS-KEY ( calcitonin* OR procalcitonin* OR pct OR "c-reactive protein" OR crp OR "molecular diagnostic techniques" ) | 393,538 |
| #9 | #5 OR #6 OR #7 OR #8 | 1,802,366 |
| #10 | TITLE-ABS-KEY ( antibiotic* OR amoxicillin* OR penicillin* OR macrolide* OR beta-lactam* OR tetracyclin* OR quinolone* OR ciprofloxacin* ) | 1,399,204 |
| #11 | TITLE-ABS-KEY ( ( anti-bacterial OR antibacterial OR bacteriocid* OR anti-infective OR antiinfective OR anti-mycobacterial OR antimycobacterial OR antimicrobial ) W/3 ( agent* OR compound* OR stewardship OR drug* OR combin* OR prescri* OR therapy OR treat* ) ) | 621,739 |
| #12 | #10 OR #11 | 1,644,349 |
| #13 | #4 AND #9 AND #12 | 18,831 |

Embase search strategy:

| Search Number | Search Details | Results |
| --- | --- | --- |
| #1 | pneumonia'/exp | 465,882 |
| #2 | pneumonia:ab,ti | 259,176 |
| #3 | cap:ab,ti | 82,123 |
| #4 | ((lung* OR pulmonary OR pleur* OR respiratory) NEAR/4 (infect* OR inflam*)):ab,ti | 249,243 |
| #5 | #1 OR #2 OR #3 OR #4 | 773,769 |
| #6 | 'point of care testing'/exp | 27,028 |
| #7 | 'point of care system'/exp | 5,206 |
| #8 | (('point of care' OR 'near patient' OR poc OR rapid OR bedside) NEAR/5 (test* OR diagnostic* OR analys* OR system* OR comput* OR immunoassay* OR techn* OR immunofluorescence OR 'fluorescent antibody' OR classif* OR detect* OR differenti* OR 'polymerase chain reaction' OR pcr OR naat OR 'molecular diagnostic*' OR 'nucleic acid amplification' OR microarray OR antigen* OR radiography OR ultraso* OR 'x ray' OR tomography)):ab,ti | 237,272 |
| #9 | 'biomarkers'/exp | 588,615 |
| #10 | biomarker*:ab,ti | 753,835 |
| #11 | (biological NEAR/3 (marker* OR indicator*)):ab,ti | 24,925 |
| #12 | 'procalcitonin'/exp | 31,958 |
| #13 | 'c reactive protein'/exp | 317,116 |
| #14 | 'molecular diagnostic techniques'/exp | 35,050 |
| #15 | calcitonin*:ab,ti OR procalcitonin*:ab,ti OR pct:ab,ti | 67,714 |
| #16 | 'c reactive protein':ab,ti OR crp:ab,ti | 225,719 |
| #17 | #6 OR #7 OR #8 OR #9 OR #10 OR #11 OR #12 OR #13 OR #14 OR #15 OR #16 | 1,574,080 |
| #18 | 'antibiotic agent'/exp | 2,176,583 |
| #19 | 'antiinfective agent'/exp | 5,610,530 |
| #20 | 'antimicrobial stewardship'/exp | 15,513 |
| #21 | 'anti-infective therapy':ab,ti OR antibiotic*:ab,ti OR amoxicillin*:ab,ti OR penicillin*:ab,ti OR macrolide*:ab,ti OR 'beta lactam*':ab,ti OR tetracyclin*:ab,ti OR quinolone*:ab,ti OR ciprofloxacin*:ab,ti | 785,139 |
| #22 | (('anti-bacterial' OR antibacterial OR bacteriocid* OR 'anti-infective' OR antiinfective OR 'anti-mycobacterial' OR antimycobacterial OR antimicrobial) NEAR/3 (agent* OR compound* OR stewardship OR drug* OR combin* OR prescri* OR therapy OR treat*)):ab,ti | 153,286 |
| #23 | #18 OR #19 OR #20 OR #21 OR #22 | 5,770,597 |
| #24 | #5 AND #17 AND #23 | 31,579 |

Cochrane Library search strategy:

| Search Number | Search Details | Results |
| --- | --- | --- |
| #1 | MeSH descriptor: [Pneumonia] explode all trees | 13,677 |
| #2 | (pneumonia):ti,ab,kw | 22,299 |
| #3 | (CAP):ti,ab,kw | 6,253 |
| #4 | (( lung* OR pulmonary OR pleur* OR respiratory ) NEAR/4 ( infect* OR inflam* )):ti,ab,kw | 22,269 |
| #5 | #1 OR #2 OR #3 OR #4 | 53,556 |
| #6 | MeSH descriptor: [Point-of-Care Testing] explode all trees | 211 |
| #7 | MeSH descriptor: [Point-of-Care Systems] explode all trees | 858 |
| #8 | (("point of care" OR "near patient" OR poc OR rapid OR bedside) NEAR/5 (test* OR diagnostic* OR analys* OR system* OR comput* OR immunoassay* OR techn* OR immunofluorescence OR "fluorescent antibody" OR classif* OR detect* OR differenti* OR "polymerase chain reaction" OR pcr OR naat OR "molecular diagnostic*" OR "nucleic acid amplification" OR microarray OR antigen* OR radiography OR ultraso* OR X-ray or tomography)):ti,ab,kw | 8,604 |
| #9 | MeSH descriptor: [Biomarkers] explode all trees | 31,782 |
| #10 | (biomarker*):ti,ab,kw | 61,373 |
| #11 | (biological NEAR/3 (marker* OR indicator*)):ti,ab,kw | 3,687 |
| #12 | MeSH descriptor: [Procalcitonin] explode all trees | 134 |
| #13 | (calcitonin* OR procalcitonin* OR pct):ti,ab,kw | 5,235 |
| #14 | MeSH descriptor: [C-Reactive Protein] explode all trees | 6,333 |
| #15 | (c reactive protein OR crp):ti,ab,kw | 37,026 |
| #16 | MeSH descriptor: [Molecular Diagnostic Techniques] explode all trees | 172 |
| #17 | #6 OR #7 OR #8 OR #9 OR #10 OR #11 OR #12 OR #13 OR #14 OR #15 OR #16 | 110,850 |
| #18 | MeSH descriptor: [Anti-Bacterial Agents] explode all trees | 16,894 |
| #19 | MeSH descriptor: [Anti-Infective Agents] explode all trees | 40,391 |
| #20 | MeSH descriptor: [Antimicrobial Stewardship] explode all trees | 137 |
| #21 | (antibiotic* OR amoxicillin* OR penicillin* OR macrolide* OR beta-Lactam* OR tetracyclin* OR quinolone* OR ciprofloxacin*):ti,ab,kw | 50,425 |
| #22 | (("anti bacterial" OR antibacterial OR bacteriocid* OR "anti infective" OR antiinfective OR "anti mycobacterial" OR antimycobacterial OR antimicrobial) NEAR/3 (agent* OR compound* OR stewardship OR drug* OR combin* OR prescri* OR therapy OR treat*)):ti,ab,kw | 24,340 |
| #23 | #18 OR #19 OR #20 OR #21 OR #22 | 79,836 |
| #24 | #5 AND #17 AND #23 | 1120 |

Web of Science search strategy:

| Search Number | Search Details | Results |
| --- | --- | --- |
| #1 | TS=(Pneumonia) and Preprint Citation Index (Exclude – Database) | 353,438 |
| #2 | TS=(CAP) and Preprint Citation Index (Exclude – Database) | 239,240 |
| #3 | TS=(( lung* OR pulmonary OR pleur* OR respiratory ) NEAR/4 ( infect* OR inflam* )) and Preprint Citation Index (Exclude – Database) | 338,964 |
| #4 | #1 OR #2 OR #3 and Preprint Citation Index (Exclude – Database) | 853,706 |
| #5 | TS=(("point-of-care" OR "near patient" OR poc OR rapid OR bedside) NEAR/5 (test* OR diagnostic* OR analys* OR system* OR comput* OR immunoassay* OR techn* OR immunofluORescence OR "fluorescent antibody" OR classif* OR detect* OR differenti* OR "polymerase chain reaction" OR pcr OR naat OR "molecular diagnostic*" OR "nucleic acid amplification" OR microarray OR antigen* OR radiography OR ultraso* OR X-ray OR tomography)) and Preprint Citation Index (Exclude – Database) | 394,048 |
| #6 | (TS=(biomarker*)) OR TS=(biological NEAR/3 (marker* OR indicator*)) and Preprint Citation Index (Exclude – Database) | 1,352,782 |
| #7 | TS=(calcitonin* OR procalcitonin* OR pct) and Preprint Citation Index (Exclude – Database) | 81,698 |
| #8 | TS=(c reactive protein OR crp) and Preprint Citation Index (Exclude – Database) | 352,907 |
| #9 | TS=(Molecular Diagnostic Techniques) and Preprint Citation Index (Exclude – Database) | 1,020,094 |
| #10 | #5 OR #6 OR #7 OR #8 OR #9 and Preprint Citation Index (Exclude – Database) | 2,928,530 |
| #11 | TS=(antibiotic* OR amoxicillin* OR penicillin* OR macrolide* OR beta-Lactam* OR tetracyclin* OR quinolone* OR ciprofloxacin*) and Preprint Citation Index (Exclude – Database) | 1,118,703 |
| #12 | TS=(("anti bacterial" OR antibacterial OR bacteriocid* OR "anti infective" OR antiinfective OR "anti mycobacterial" OR antimycobacterial OR antimicrobial) NEAR/3 (agent* OR compound* OR stewardship OR drug* OR combin* OR prescri* OR therapy OR treat*)) and Preprint Citation Index (Exclude – Database) | 1,129,943 |
| #13 | #11 OR #12 and Preprint Citation Index (Exclude – Database) | 1,717,615 |
| #14 | #4 AND #10 AND #13 and Preprint Citation Index (Exclude – Database) | 11,156 |

## Appendix (2): the studies included in the meta-analysis.

[1] Christ-Crain M, Jaccard-Stolz D, Bingisser R, Gencay MM, Huber PR, Tamm M, et al. Effect of procalcitonin-guided treatment on antibiotic use and outcome in lower respiratory tract infections: cluster-randomised, single-blinded intervention trial. *Lancet* 2004;363;9409:600-7.doi:10.1016/s0140-6736(04)15591-8.

[2] Huang DT, Yealy DM, Filbin MR, Brown AM, Chang CH, Doi Y, et al. Procalcitonin-Guided Use of Antibiotics for Lower Respiratory Tract Infection. *N Engl J Med* 2018;379;3:236-49.doi:10.1056/NEJMoa1802670.

[3] Schuetz P, Christ-Crain M, Thomann R, Falconnier C, Wolbers M, Widmer I, et al. Effect of procalcitonin-based guidelines vs standard guidelines on antibiotic use in lower respiratory tract infections: the ProHOSP randomized controlled trial. *Jama* 2009;302;10:1059-66.doi:10.1001/jama.2009.1297.

[4] Shengchen D, Gu X, Fan G, Sun R, Wang Y, Yu D, et al. Evaluation of a molecular point-of-care testing for viral and atypical pathogens on intravenous antibiotic duration in hospitalized adults with lower respiratory tract infection: a randomized clinical trial. *Clin Microbiol Infect* 2019;25;11:1415-21.doi:10.1016/j.cmi.2019.06.012.

[5] Cartuliares MB, Rosenvinge FS, Mogensen CB, Skovsted TA, Andersen SL, Østergaard C, et al. Evaluation of point-of-care multiplex polymerase chain reaction in guiding antibiotic treatment of patients acutely admitted with suspected community-acquired pneumonia in Denmark: A multicentre randomised controlled trial. *PLoS Med* 2023;20;11:e1004314.doi:10.1371/journal.pmed.1004314.

[6] Christ-Crain M, Stolz D, Bingisser R, Müller C, Miedinger D, Huber PR, et al. Procalcitonin guidance of antibiotic therapy in community-acquired pneumonia: a randomized trial. *Am J Respir Crit Care Med* 2006;174;1:84-93.doi:10.1164/rccm.200512-1922OC.

[7] Long W, Deng X, Zhang Y, Lu G, Xie J, Tang J. Procalcitonin guidance for reduction of antibiotic use in low-risk outpatients with community-acquired pneumonia. *Respirology* 2011;16;5:819-24.doi:10.1111/j.1440-1843.2011.01978.x.

[8] Montassier E, Javaudin F, Moustafa F, Nandjou D, Maignan M, Hardouin JB, et al. Guideline-Based Clinical Assessment Versus Procalcitonin-Guided Antibiotic Use in Pneumonia: A Pragmatic Randomized Trial. *Ann Emerg Med* 2019;74;4:580-91.doi:10.1016/j.annemergmed.2019.02.025.

[9] Briel M, Schuetz P, Mueller B, Young J, Schild U, Nusbaumer C, et al. Procalcitonin-guided antibiotic use vs a standard approach for acute respiratory tract infections in primary care. *Arch Intern Med* 2008;168;18:2000-7; discussion 7-8.doi:10.1001/archinte.168.18.2000.

## Appendix (3): Characteristics of each included study.

| **Study author** | **Country** | **Setting** | **Sample size (POCT/usual care)** | **Disease classification** | **the type of POCT** | **Testing of POCT** | **POCT algorithm used** |
| --- | --- | --- | --- | --- | --- | --- | --- |
| Christ et al, 2004 | Switzerland | Hospital | 87 (42/45) | LRTI including CAP | PCT | 5min-2h | A procalcitonin value of 0.1–0.25 μg/L was regarded as an indication that bacterial infection was unlikely, and we discouraged use of antibiotics. We deemed serum procalcitonin between 0.25 and 0.5 μg/L to indicate a possible bacterial infection, and the treating doctor was advised to initiate antimicrobial treatment. A procalcitonin value of 0.5 μg/L or greater was judged suggestive of the presence of bacterial infection, and we strongly recommended antibiotic treatment. |
| Christ et al, 2006 | Switzerland | Hospital | 302 (151/151) | CAP | PCT | 5min-2h | A procalcitonin level of less than 0.1 g/L suggested the absence of bacterial infection and the initiation or continuation of antibiotics was strongly discouraged. A procalcitonin level between 0.1 and 0.25 g/L indicated that bacterial infection was unlikely, and the initiation or continuation of antibiotics was discouraged. A procalcitonin level from 0.25 to 0.5 g/L was considered to indicate a possible bacterial infection and the initiation or continuation of antibiotic therapy was encouraged. A procalcitonin level greater than 0.5 g/L strongly suggested the presence of bacterial infection and antibiotic treatment and continuation was strongly encouraged. |
| Briel et al, 2008 | Switzerland | Hospital | 69 (38/31) | ARI including CAP | PCT | 2-4h | In patients with PCT levels lower than 0.1 μg/L, a bacterial infection was considered highly unlikely, and the use of antibiotics was discouraged. In patients with a PCT level higher than 0.25 μg/L, a bacterial infection was considered likely and the use of antibiotics was recommended. For PCT concentrations of 0.1 to 0.25 μg/L, a bacterial infection was considered unlikely, and the use of antibiotics was not recommended. |
| Schuetz et al, 2009 | Switzerland | Hospital | 925 (460/465) | LRTI including CAP | PCT | 5min-2h | Initiation or continuation of antibiotics was strongly discouraged if PCT was less than 0.1 μg/L and discouraged if levels were 0.25 μg/L or lower. Initiation or continuation of antibiotics was strongly encouraged if PCT was higher than 0.5 μg/L and encouraged if levels were higher than 0.25 μg/L |
| Long et al, 2011 | China | Hospital | 156 (77/79) | CAP | PCT | 5min-2h | A PCT level <0.1 mg/L suggested the absence of bacterial infection and the initiation or continuation of antibiotic treatment was strongly discouraged. A PCT level of 0.1–0.25 mg/L indicated that bacterial infection was unlikely, and the initiation or continuation of antibiotic treatment was discouraged. A PCT level >0.25 mg/L was considered to indicate a possible bacterial infection and the initiation or continuation of antibiotic therapy was encouraged. |
| Huang et al , 2018 | United States | Hospital | 328 (167/161) | LRTI including CAP | PCT | 5min-2h | With antibiotics strongly discouraged for procalcitonin levels <0.1 μg per liter, discouraged for levels 0.1 to 0.25 μg per liter, recommended for levels >0.25 to 0.5 μg per liter, and strongly recommended for levels >0.5 μg per liter |
| Montassier et al, 2019 | France | Hospital | 285 (142/143) | CAP | PCT | 5min-2h | Initiation of antibiotics was strongly not recommended if the procalcitonin level was less than 0.1 mg/L and was not recommended if the level was less than or equal to 0.25 mg/L; and initiation of antibiotics was strongly recommended if the procalcitonin level was greater than 0.5 mg/L and recommended if the level was greater than 0.25 mg/L. |
| Shengchen et al, 2019 | China | Hospital | 456 (242/214) | LRTI including CAP | Molecular POCT | 5min-2h | FilmArray Respiratory Panel (BioFire; Salt Lake City, UT, USA) is a new molecular point-of-care test (POCT) platform, which can simultaneously detect 20 viruses and atypical pathogens and provide results in about 1 hour |
| Bichuette Cartuliares et al, 2023 | Denmark | Hospital | 291 (145/146) | CAP | Molecular POCT | 5min-2h | The Biofire FilmArray Pneumonia Panel plus (Biome ́rieux, Marcy l’Etoile, France) is an automatic, closed, multiplex PCR, that includes all steps of molecular diagnostics in about 75 min, including sample preparation. The panel detects 18 bacterial pathogens, 9 viruses, and 7 antimicrobial resistance genes |

## Appendix (4): Risk of bias graph.

## Appendix (5): Sensitivity analysis using risk ratio for all outcomes.

|  | Studies  (n) | Participants  (n) | RR  (95% CI) | *I²* (%) | *p-value* |
| --- | --- | --- | --- | --- | --- |
| **Antibiotic prescription rate** |  |  |  |  |  |
| Overall result | 9 | 2899 | 0.91 (0.86, 0.95)^a^ | 79 | <0.001 |
| Type of POCT |  |  |  |  |  |
| PCT | 7 | 2152 | 0.89 (0.85, 0.93)^a^ | 60 | <0.001 |
| Molecular POCT | 2 | 747 | 1.00 (0.84, 1.10)^a^ | 0 | 0.80 |
| **ICU transfer rate** |  |  |  |  |  |
| Overall result | 3 | 878 | 1.08 (0.71, 1.65) | 32 | 0.71 |
| Type of POCT |  |  |  |  |  |
| PCT | 2 | 587 | 1.19 (0.77, 1.85) | 28 | 0.44 |
| Molecular POCT | 1 | 291 | 0.40 (0.08, 2.04) | NA | 0.27 |
| **30-day all-cause mortality** |  |  |  |  |  |
| Overall result | 3 | 1501 | 1.01 (0.36, 2.83) | 0 | 0.83 |
| Type of POCT |  |  |  |  |  |
| PCT | 2 | 1210 | 0.91 (0.54, 1.52) | 0 | 0.71 |
| Molecular POCT | 1 | 291 | 1.26 (0.34, 4.59) | NA | 0.73 |
| **30-day all-cause readmission** | 1 | 291 | 0.86 (0.47, 1.57) | NA | 0.61 |
| **Composite adverse events rate** |  |  |  |  |  |
| Overall result | 3 | 1501 | 0.74 (0.62, 0.88) | 0 | <0.001 |
| Type of POCT |  |  |  |  |  |
| PCT | 2 | 1210 | 0.72 (0.59, 0.87) | 0 | <0.001 |
| Molecular POCT | 1 | 291 | 0.85 (0.54, 1.34) | NA | 0.49 |

Notes: CI = confidence interval. NA = not available. NR = not reported. RR = risk ratio. MD = mean difference. SMD = standardized mean difference. a refers to RR; b refers to MD. c refers to SMD
